# New Work on Materia Medica and Therapeutics

**Published:** 1852-11

**Authors:** 


*New Work on Materia Medica and Therapeutics.*—The Boston Med. and Surg. Journal states that it is proposed to publish, by subscription, a work on materia medica and therapeutics, by Prof. W. Tully, of New Haven, Ct. Our cotemporary enumerates the peculiar features of the work as follows: "*First.* It will be original, having none of the characteristics of a compilation. *Second.* It will contain a large amount of practical information not found in ordinary books on the subject. *Third.* The powers and operations of medicines will be described with minuteness and precision. *Fourth.* The doctrines it inculcates are not speculative or theoretical, but eminently practical. *Fifth.* It will be written with ability and learning, such as would do credit to the profession in any country." The plan of publication is to issue the work in numbers, 25 cents each—four, at least, to be subscribed for at one time. It will be printed in double columns, with good type and paper, and in the best style. The Nos. will be issued on the first of every month, commencing in November. Subscriptions will be received by Dr. J. Church, Springfield, Mass.

